# The role of the hyaline spheres in sea cucumber metamorphosis: lipid storage via transport cells in the blastocoel

**DOI:** 10.1186/s13227-019-0119-4

**Published:** 2019-04-11

**Authors:** Josefina Peters-Didier, Mary A. Sewell

**Affiliations:** School of Biological Sciences, University of Auckland, Private Bag 92019, Auckland, 1142 New Zealand

**Keywords:** Sea cucumber, Metamorphosis, Hyaline spheres, Holothuroidea, Lipid utilization

## Abstract

**Background:**

For echinoderms with feeding larvae, metamorphic and post-settlement success may be highly dependent on larval nutrition and the accumulation of energetic lipids from the diet. In contrast to the sea urchins, starfish and brittle stars within the Phylum Echinodermata, sea cucumber metamorphosis does not involve formation of a juvenile rudiment, but instead there is a rearrangement of the entire larval body. Successful metamorphosis in sea cucumbers is often associated with the presence in the late auricularia stage of an evolutionary novelty, the hyaline spheres (HS), which form in the base of the larval arms. Known since the 1850s the function of these HS has remained enigmatic—suggestions include assistance with flotation, as an organizer for ciliary band formation during metamorphosis and as a nutrient store for metamorphosis.

**Results:**

Here using multiple methodologies (lipid mapping, resin-section light microscopy, lipid and fatty acid analyses) we show definitively that the HS are used to store neutral lipids that fuel the process of metamorphosis in *Australostichopus mollis*. Neutral lipids derived from the phytoplankton diet are transported by secondary mesenchyme cells (“lipid transporting cells”, LTC), likely as free fatty acids or lipoproteins, from the walls of the stomach and intestine through the blastocoel to the HS; here, they are converted to triacylglycerol with a higher saturated fatty acid content. During metamorphosis the HS decreased in size as the triacylglycerol was consumed and LTC again transported neutral lipids within the blastocoel.

**Conclusion:**

The HS *in A. mollis* functions as a nutrient storage structure that separates lipid stores from the major morphogenic events that occur during the metamorphic transition from auricularia–doliolaria–pentactula (settled juvenile). The discovery of LTC within the blastocoel of sea cucumbers has implications for other invertebrate larvae with a gel-filled blastocoel and for our understanding of lipid use during metamorphosis in marine invertebrates.

## Background

The echinoderms, basal members of the Deuterostomia, are well known in an evo-devo context because of the presence of morphologically distinct larval forms in each echinoderm class [1, 2]. In species with feeding larvae, metamorphic and post-settlement success may be highly dependent on larval nutrition and the accumulation of energetic lipids from the diet [3–5]. These lipid reserves, stored in echinoids and asteroids in the epithelium of the larval stomach [3, 4, 6], fuel juvenile development until the juvenile can feed on its own, and the amounts stored prior to metamorphosis can have later impacts on juvenile survival and adult performance [6]. Detailed study of lipid dynamics during development in echinoids and asteroids has shown that neutral lipids, such as triacylglycerol (TAG) and free fatty acids (FFA), are stored during the feeding period to fuel the process of metamorphosis [3, 4].

Holothuroids are the echinoderm class that most fully represents the ancestral type of indirect larval development: a feeding dipleurula-type larva (the auricularia) followed by a non-feeding doliolaria [1, 2, 8]. Metamorphosis in holothuroids also differs from other echinoderm classes in that a juvenile rudiment is not formed, but instead metamorphosis involves a series of morphogenic movements that reorganize most of the larval structures (with the exception of the larval gut) to become the juvenile structures [2, 7–9]. The lack of a distinct metamorphosis in holothurians and the confluence of larval and adult body plans in this class have led some to suggest that this is a paedomorphic condition [2, 10, 11].

Successful metamorphosis in sea cucumbers is often associated with the presence in the late auricularia stage of enigmatic structures, the hyaline spheres (HS), which form in the larval arms during the feeding phase, increase in diameter in the late auricularia before metamorphosis into the doliolaria stage and then decrease in size during the pentactula and juvenile stage [7–10, 12–16]. HS were first described in the 1850s by Müller [17] as “elastiche Kugel”; this term was also used by Hörstadius [18], and in later publications in English as “elastic balls” [12] and “spheres” [13]. HS appear in species from three clades in the recent molecular phylogeny of the Holothuroidea [19], in the families Holothuriidae, Stichopodidae and Synaptidae, but are absent in crinoids and echinoderm classes that form a rudiment during metamorphosis [7].

Mortensen [12] first hypothesized that HS had a role during metamorphosis, although they have also been suggested to assist with flotation [12, 13], as an organizer for ciliary band formation in the doliolaria [20], or that their function is related to larval locomotion [21]. Indirect evidence has accumulated that HS have a nutritional role in metamorphosis: HS are more common in late auricularia fed with a mixed diet [7, 14, 22]; they presage the successful and rapid completion of metamorphosis [23], a shorter larval duration and increased juvenile survival [14]. Staining with Sudan dyes has also suggested that HS contain lipid [14, 15], but this staining pattern may be an artefact of the gel-like nature of the HS [16]. Lacking to date is any direct evidence for the role of HS as a nutritional store for metamorphosis, or a mechanism whereby nutrients could be transported from the larval digestive system to the HS [7, 15, 16].

Here, using auricularia larvae of the New Zealand sea cucumber *Australostichopus mollis* we directly test the hypothesis of a nutritional function for the HS using lipid mapping, resin-section light microscopy and lipid and fatty acid analyses. Our previous work has shown that, like asteroids and echinoids, *A. mollis* use the neutral lipids TAG and FFA to build the larval body [24], and we predicted that, as in other echinoderms [3, 4], these lipids would also be used to fuel metamorphosis. Consequently, our approach to study the HS was to use specific dyes to stain lipids—firstly the lipophilic Nile Red and then using more specific neutral lipid and phospholipid fluorescent probes. We focused in detail on the mechanism through which lipids are transported to the HS, as although HS are an evolutionary novelty, it is likely that mechanisms of lipid transport may be a shared feature of larvae in other echinoderm classes and hemichordates with a gel-filled blastocoel.

## Results

### Late auricularia

**Fig. 1:**
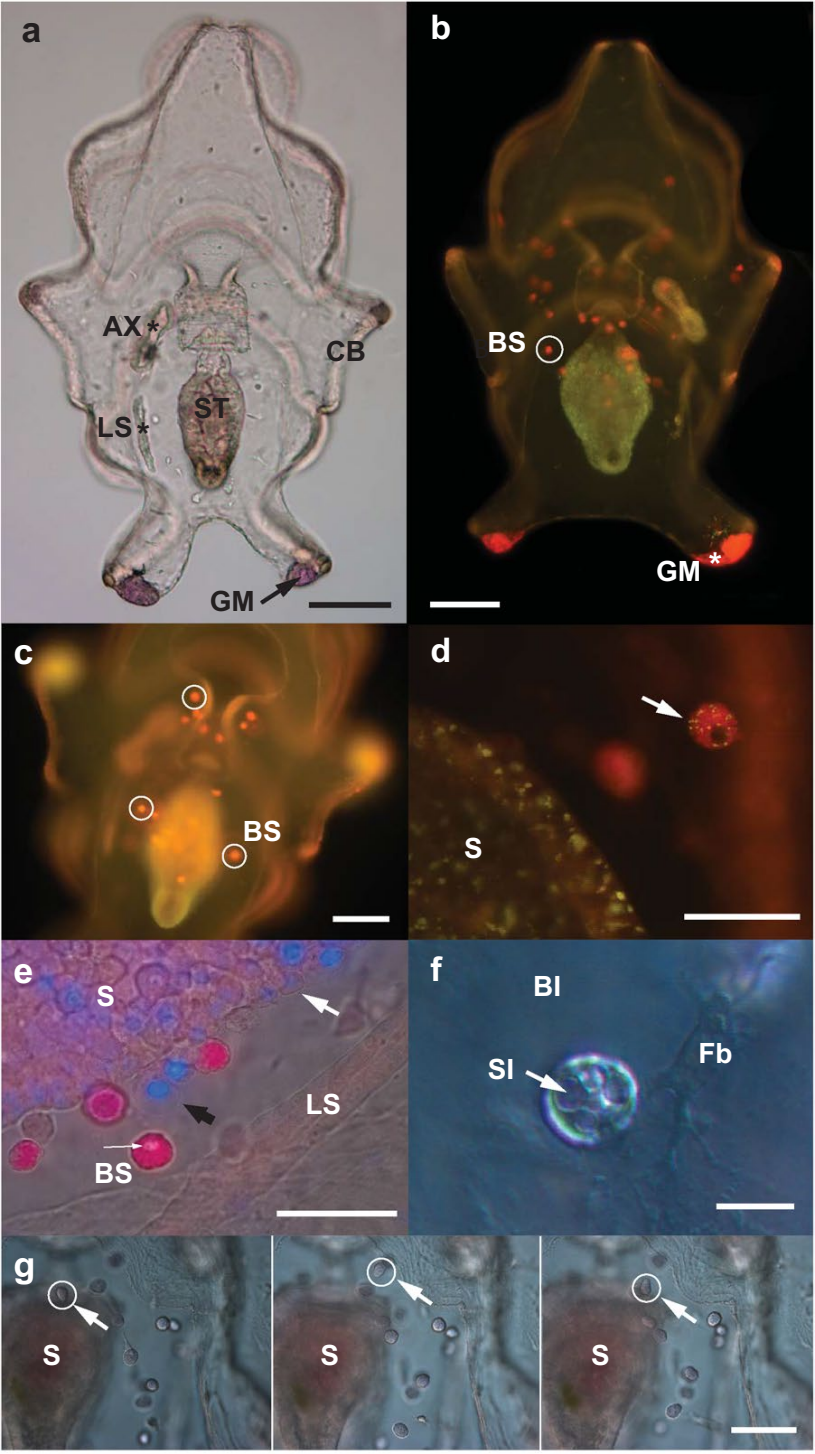
Late auricularia of *A. mollis*. **a** Light microscope view of general anatomy of 14-day larva showing ciliated band (CB), stomach (ST), axohydrocoel (AX, position shown by *), left somatocoel (LS, position shown by *) and granular mass (GM). Scale bar 100 µm. **b** 14-day larva stained with Nile Red under blue light excitation and polarized light. Stomach fluoresces green; one of many red blastocoel spherules (BS) shown with white circle; granular mass (GM, position shown by *) also fluoresces red. Scale bar 100 µm. **c** 16-day auricularia stained with Nile Red under blue light excitation. Some blastocoel spherules (BS) are highlighted with white circles. Scale bar 200 µm. **d** 16-day auricularia stained with Nile Red under blue light excitation. Stomach (S) and blastocoelic spherule (arrow) carrying both neutral lipids (yellow) and more polar lipids (red). Scale bar 25 µm. **e** 16-day auricularia larvae stained with Nile Red and Hoechst nuclear stain under simultaneous blue light excitation and light microscopy. Stomach (S), left somatocoel (LS) and blastocoelic spherule (BS), with likely location of the nucleus (thin arrow). Black arrow indicates a cell of similar size to the BS that appears to differentiate from the stomach epithelium. White arrow indicates a smaller cell type lining the stomach epithelium. Scale bar: 25 µm. **f** Blastocoelic spherule within the blastocoel (Bl) of a 14-day larva stained with Nile Red under Nomarski light microscopy. Spherical inclusions (SI) in the blastocoelic spherule are indicated by arrow. Blastocoel also contains a fibroblast-type cell (Fb). Scale bar: 12.5 µm. **g** Blastocoelic spherule amoeboid movement in a 14-day auricularia larva stained with Nile Red under light microscopy. Arrows indicate sequence of movement of one blastocoelic spherule (white arrow, white circle) in a time lapse of 40 min. Scale bar: 50 µm

Larvae of the sea cucumber *Australostichopus mollis* reach the late auricularia stage approximately 14 days after fertilization, defined as when the left somatocoel was about half the length of the stomach and there was no elongation of the axohydrocoel (Fig. 1a). Staining with lipophilic Nile Red revealed an accumulation of neutral lipids (green-yellow) in the stomach epithelium and multiple spherical structures ~ 10 µm in diameter suspended in the larval blastocoel which fluoresced red, indicating they contained more polar lipids or lipoproteins (Fig. 1b). These blastocoelic spherules (BS) were transparent, had circular inclusions and developed in close association with the digestive system (Fig. 1b–f). BS were very labile and continuously changed form and surface texture from smooth to coarse. Sequential photographic images revealed the BS were capable of independent and active amoeboid movement through the larval blastocoel (Fig. 1g).

The strong red fluorescence of Nile Red-stained BS indicated they possibly contain phospholipids, amphipathic lipids or hydrophobic proteins [25, 26], although some BS also contained traces of yellow-staining neutral lipids (Fig. 1d). Counterstaining with Hoechst 33342 revealed a fluorescent nucleus only in the early stages of BS formation; the nuclear dye did not seem to be able to penetrate fully formed BS, although a non-fluorescent spot indicated the likely location of the nucleus (Fig. 1e).

**Fig. 2:**
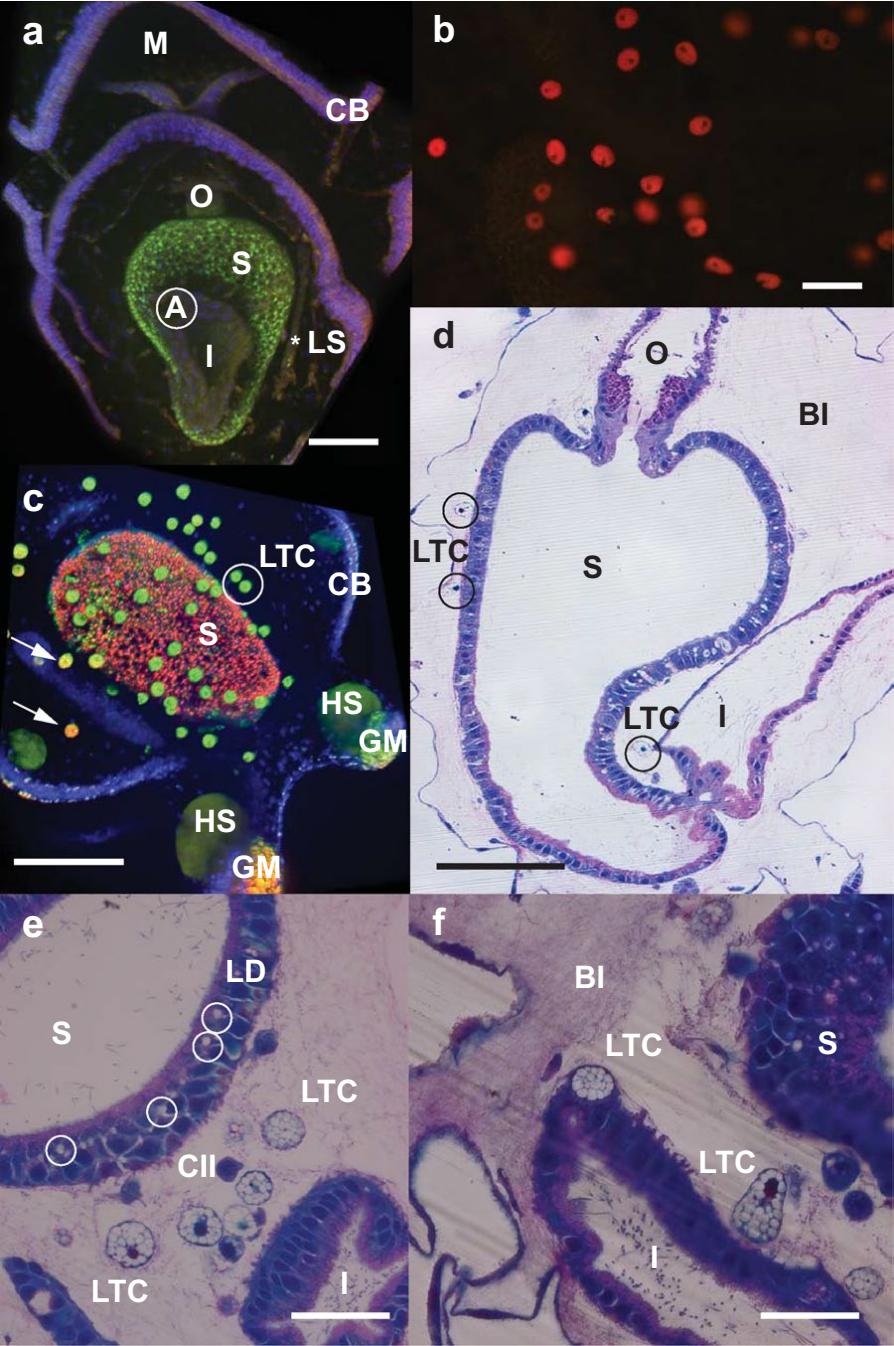
Late auricularia of *A. mollis*. **a** Confocal image of 14-day auricularia stained with Hoechst nuclear dye and LipidTOX™ green neutral lipid stain. Full digestive system is shown: mouth (M), oesophagus (O), stomach (S), intestine (I), anus (A). Only stomach stains green for neutral lipids. Ciliary band (CB) and left somatocoel (LS, position shown by *) are also shown. Scale bar: 50 µm. **b** 16-day auricularia stained with Nile Red under blue light excitation showing multiple red-staining LTC within the blastocoel. Scale bar: 25 µm. **c** Confocal image of 20-day auricularia stained with Hoechst nuclear dye, LipidTOX™ green neutral lipid stain and LipidTOX™ red phospholipid stain. Lipid transporting cells (LTC, white circle) and hyaline spheres (HS) stain green for neutral lipid; some LTC (white arrows) and one granular mass (GM) show mixed fluorescence between green and red. Ciliary band (CB) and stomach (S) are also shown. Scale bar: 100 µm. **d** Longitudinal section of digestive system of 14-day auricularia. Oesophagus (O), stomach (S), intestine (I), blastocoel (Bl), lipid transporting cell (LTC, black circle). Collagen/elastin fibres are present in the blastocoel. Scale bar: 60 µm. **e** Longitudinal section through 16-day auricularia. Lipid transport cells (LTC) and progenitor type of cells (CII) suspended in the blastocoel. Stomach (S) and intestine (I) are both lined internally with pink-staining mucus layer. Lipid deposits (LD) in the stomach wall are circled (white circle). Scale bar: 25 µm. **f** LTC emerging from the digestive epithelium of the intestine (I) and into the blastocoelic space (Bl) between the stomach (S) and the intestine. Blastocoelic space is filled with collagen/elastin fibres. Scale bar: 25 µm

Plastic sections revealed at least three cell types within the blastocoelic matrix: (1) a ~ 5-µm-diameter, slightly flattened fibroblast-type cell with a clear nucleus and blue-staining cytoplasm with variable degree and number of cell projections (Fig. 1f); (2) a ~ 5-µm rounder cell with a large nucleus and reduced blue-staining cytoplasm (Fig. 2e, CII); and (3) a ~ 10-µm cell with a clear eccentrically positioned nucleus, usually spherical but also showing multiple spheroidal shapes (Fig. 2e, f). This third cell type contained well-delineated empty spaces within the cell cytoplasm and traces of green/grey staining which, with the methylene blue-azure II and basic fuchsine stain used, likely corresponded to intracellular lipid droplets (Fig. 2e, f). As the diameter of this third type of cell matched the diameter of the BS observed suspended in the blastocoel of live larvae, and both contained conspicuous spherical inclusions of apparent lipid nature, we infer that they are the same type of cell and now refer to them as lipid transporting cells (LTC).

To address the origin of the LTC we focused attention on the digestive tract of the late auricularia, which showed different epithelial cell morphologies along its length: cuboidal epithelial cells in the oesophagus, columnar epithelial tissue with ciliated cells in the stomach, and squamous and cuboidal epithelial tissue with ciliated cells in the intestine (Fig. 2b). In more advanced late auricularia (approximately 16 days) lipid droplets were evident in the stomach wall, in the LTC that were closely associated with the stomach wall (Fig. 2e) and in LTC within the blastocoelic gel matrix (Fig. 2b, e, f). LTC appeared to originate and differentiate from the epithelium of the stomach and intestine, becoming freely moving inside the larval blastocoelic space (Figs. 1g, 2e, f), increasing in number and moving distally from the digestive tract as development proceeded (Fig. 2b).

**Fig. 3:**
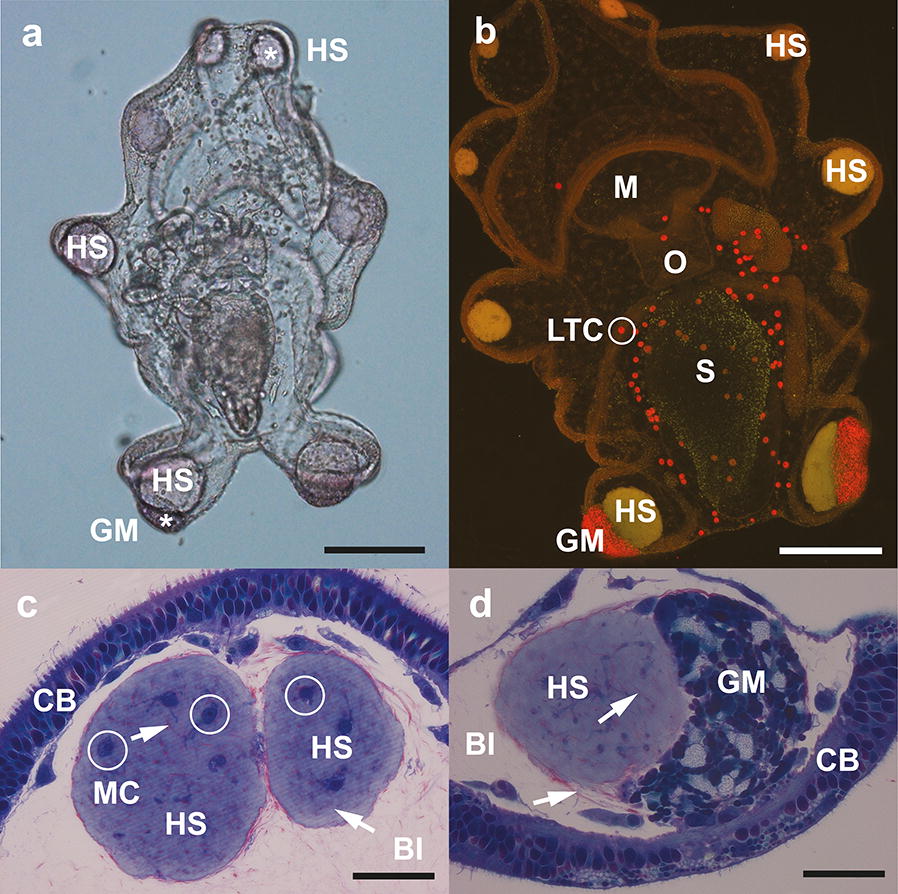
Late auricularia of *A. mollis* with detail of hyaline spheres. **a** Light microscope image of 21-day auricularia showing numerous hyaline spheres (HS and *) and granular mass (GM, position shown by *) in the posterolateral larval lobes. Scale bar: 200 µm. **b** 19-day auricularia stained with Nile Red under blue light excitation; larva gently pressed against the glass slide using a coverslip. Hyaline spheres (HS) in all lobes stain yellow for neutral lipid. Lipid transporting cells (LTC; white circle) and granular mass (GM) stain red for a more polar lipid. Digestive system includes mouth (M), oesophagus (O), stomach (S). Scale bar: 200 µm. **c** Section through a hyaline sphere (HS) within blastocoel (Bl), adjacent to the ciliated band (CB) of 16-day auricularia. Macrophage-type cells (MC, white circle) are present within the HS. Arrow indicates collagen/elastin fibres inside the HS. Scale bar: 25 µm. **d** Hyaline sphere (HS) next to a granular mass (GM), abutting the ciliated band (CB) in one of the posterolateral larval lobes of 16-day auricularia. Arrows indicate collagen/elastin fibres in the blastocoel (Bl) and inside the HS. Scale bar: 25 µm

LipidTOX™ green neutral lipid stain confirmed that neutral lipids in late auricularia (14 days) were strongly concentrated in the stomach (Fig. 2a). In later stage larvae (20 days), the neutral lipids appeared to be transferred to the LTC; the stomach fluoresced mostly red for phospholipids using LipidTOX™ red phospholipid stain (Fig. 2c). The incongruity of the LTC containing a dense aggregation of neutral lipids when stained with the specific neutral LipidTOX™ and not phospholipids as indicated using Nile Red (Figs. 1b–d, 2b, 3a) indicates that LTC might transport a more polar type of neutral lipid, a hypothesis that we address later in the Results section.

### Hyaline sphere formation

Early-stage auricularia developed two granular masses, similar to those described by Semon [24] and Mortensen [12] in the two posterolateral arms (Fig. 3a). The granular masses (GM) developed in conjunction with the larval skeletal ossicle and measured ~ 50 µm diameter, indicated polar lipids with Nile Red and appeared to be composed of scattered cells inside an extracellular matrix (Fig. 1a, b). Plastic sections revealed that GM consisted of a heterogeneous matrix of cells, connective tissue and what appeared to be packed and inactive LTC containing numerous small lipid droplets (Fig. 3c). GM mostly fluoresced green for neutral lipids with LipidTOX™ (Fig. 2c), after initial red fluorescence with Nile Red indicated they contained more polar compounds (Fig. 1b, 3b); a pattern previously seen in the LTC as discussed above.

At about 19 days post-fertilization, hyaline spheres (HS) started forming in the posterolateral lobes of the late auricularia larvae, just above the GM (Figs. 2c, 3a). HS fluoresced intensely indicating neutral lipids (yellow for Nile Red; green for LipidTOX™ (Figs. 2c, 3b). A minority of LTC fluoresced towards the red spectrum, suggesting the LTC might also transport neutral lipids of a more polar nature than those accumulating in the HS (Fig. 2c).

Towards 20 days post-fertilization, more HS began appearing sequentially in the lateral and anterior larval lobes, reaching a maximum number of 10 per larva and increasing in size as development proceeded (Fig. 3a). In light microscopy, the HS had a refractile appearance, making them easily distinguishable from the two more opaque GM located in the posterolateral lobes (Fig. 3a). In plastic sections, the HS consisted of an extracellular matrix of homogeneous material that stained light grey and contained embedded fibres and a few small macrophage-type cells (Fig. 3c, d). All HS, including those associated with the GM, were surrounded by fibres within the blastocoel; these fibres held the HS very close to the lateral lobes of the larvae, but without an apparent connection to the ciliary band (Fig. 3c, d).

### Doliolaria transition and metamorphosis

**Fig. 4:**
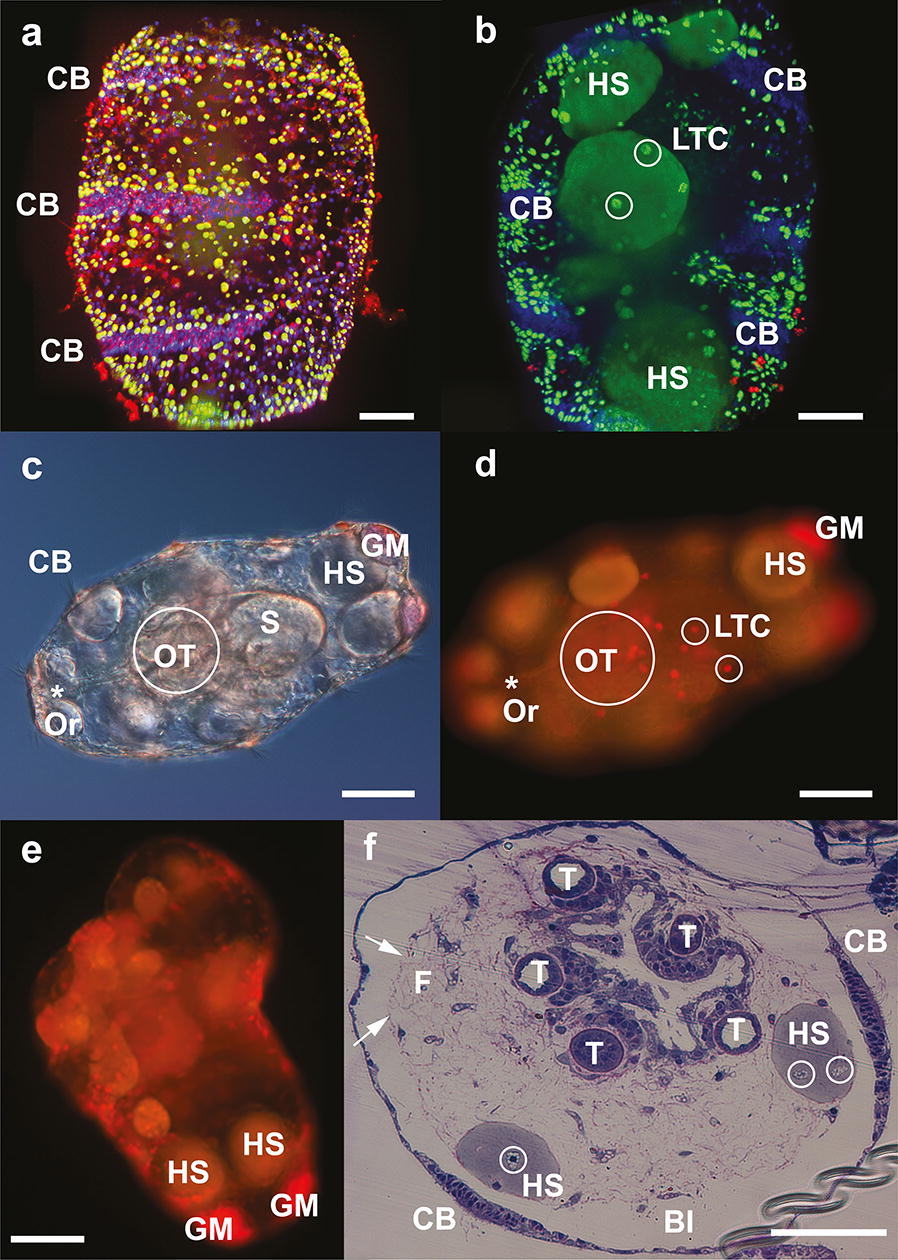
Doliolaria of *A. mollis*. **a** Confocal image showing outer structure with three ciliated bands (CB) of 23-day doliolaria stained with Hoechst nuclear dye, LipidTOX™ green neutral lipid stain and LipidTOX™ red phospholipid stain. Scale bar: 50 µm. **b** Inner structures of same larvae as in Panel A highlighting ciliated band (CB), hyaline spheres (HS) and lipid transporting cells (LTC, white circle). Scale bar: 50 µm. **c** Doliolaria larva (23-day) stained with Nile Red under Nomarski light microscopy with ciliated band (CB), stomach (S), hyaline spheres (HS), granular mass (GM), area containing the 5 oral tentacles (OT, white circle), and new oral opening (Or, position shown by *). Scale bar: 100 µm. **d** Same larva as in Panel C under blue light excitation highlighting lipid transporting cells (LTC, white circle). Scale bar: 100 µm. **e** 25-day doliolaria larva with protruding oral tentacles stained with Nile Red under blue light excitation. Hyaline spheres (HS), granular mass (GM). Scale bar: 100 µm. **f** Section through 23-day doliolaria larva. Blastocoel (Bl); ciliary band (CB); hyaline spheres (HS) containing lipid transporting cells (white circle); oral tentacles (T); fibrillar matrix (F). Arrows indicate collagen/elastin fibres in the blastocoel pulling the HS towards the centre of the larva. Scale bar: 100 µm

During transition from the late auricularia to the doliolaria (approximately 22–23 days) the continuous ciliary band broke to form three ciliary rings (Fig. 4a). The central mouth closed and reopened on the frontal side of the larvae (Fig. 4c, d), the larvae dramatically decreased in size, and oral tentacle formation was evident (Fig. 4c, d). Larvae lost transparency during this metamorphic process, so detailed study of events occurring inside the larvae required the use of lipid-specific dyes or histological sections.

All the doliolaria observed in culture had the blastocoel filled with HS and LTC; the latter were more abundant distally from the digestive tract (Fig. 4a, d). Granular masses were still evident in the posterior of the doliolaria below the HS (Fig. 4e). Fibres in the blastocoelic space became denser and seemed to aggregate and pull the HS away from the larval body wall towards the digestive system in the centre of the larva (Fig. 4f). Unlike the auricularia larvae, the macrophage-type cell was not observed inside the HS; in their place, LTC were encountered (Fig. 4f).

**Fig. 5:**
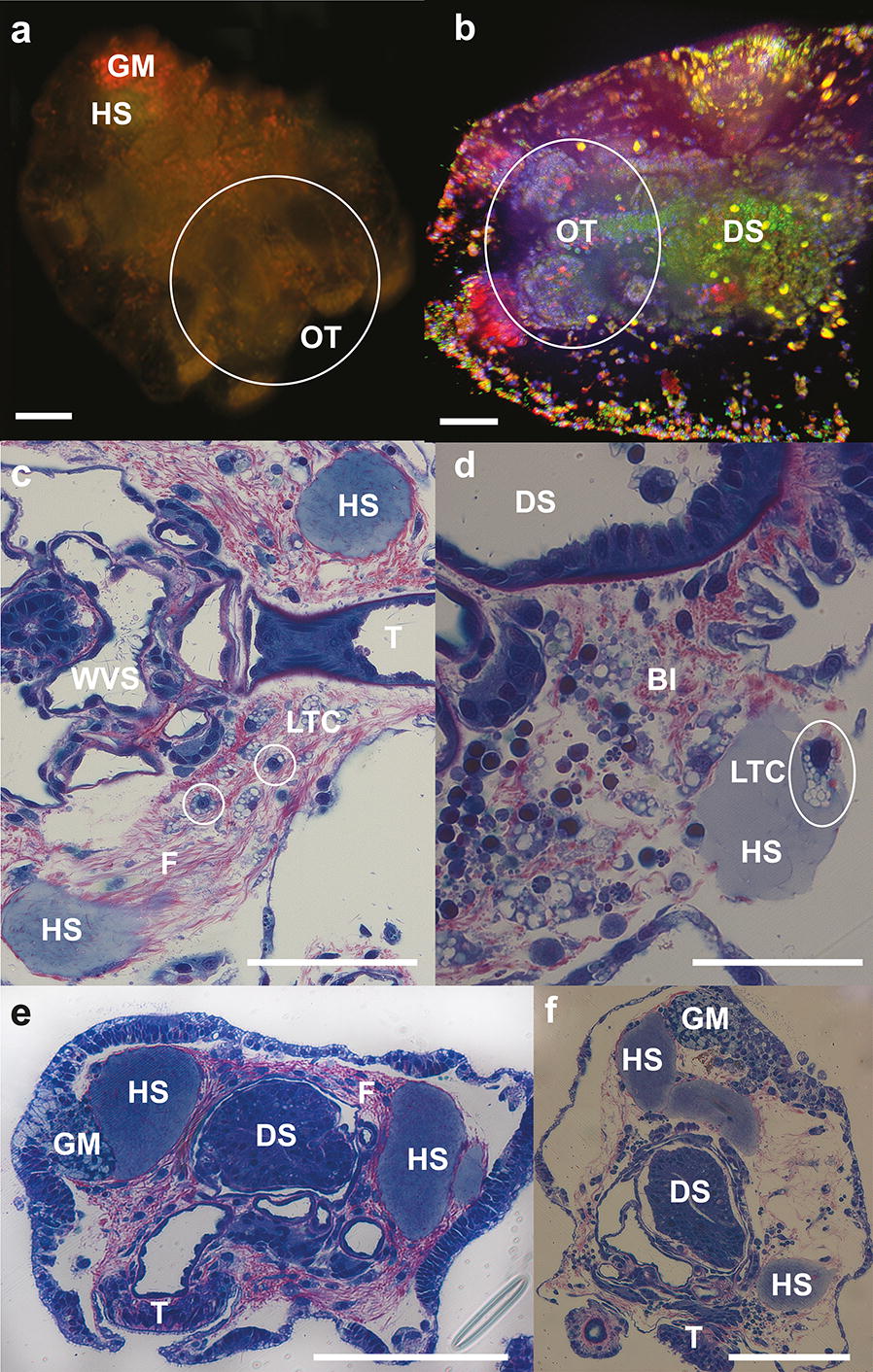
*A. mollis* pentactula (early juvenile). **a** Pentactula (27-day) stained with Nile Red under blue light excitation. Note minimal external lipid staining. Granular mass (GM), hyaline spheres (HS), oral tentacles (OT, circled). Scale bar: 75 µm. **b** Confocal image of pentactula (27-day) stained with Hoechst nuclear dye, LipidTOX™ green neutral lipid stain and LipidTOX™ red phospholipid stain showing formation of juvenile digestive system (DS) and oral tentacles (OT, circled). Scale bar: 50 µm. **c** Section through pentactula (27-day) with degrading hyaline spheres (HS) and multiple lipid transporting cells (LTC, white circle) between blastocoelic fibres (F). Oral tentacles (T) and water vascular system (WVS). Scale bar: 50 µm. **d** Pentactula (27-day) with degrading hyaline spheres (HS), developing juvenile digestive system (DS) and lipid transporting cells (LTC, white circle) in blastocoel (Bl) and within the HS. Scale bar: 35 µm. **e** Pentactula (27-day) longitudinal section showing hyaline spheres (HS) close to developing juvenile digestive system (DS). Oral tentacles (T), fibrillar matrix (F) and granular mass (GM). Scale bar: 80 μm. **f** Pentactula (27-day) longitudinal section showing hyaline spheres (HS) close to developing juvenile digestive system (DS). Oral tentacles (T), granular mass (GM). Scale bar: 120 μm

In the recently settled early pentactula (27 days), blastocoelic fibres were tightly packed in the centre of the juvenile body, holding the HS close to the digestive system but allowing transit of different cell types within the fibre matrix (Fig. 5c, d). LTC were abundant, especially inside the HS, and in the blastocoelic space and the HS appeared to start breaking apart and reducing in size (Fig. 5c, d). Pentactula showed little evidence of Nile Red staining (Fig. 5a) due to the attainment of juvenile pigments and numerous table-shaped ossicles that protruded from the body wall. After consumption of the HS, neutral lipids were mainly concentrated around the stomach and the body wall (Fig. 5a, b).

### Identification of the lipid in the HS and LTC

**Fig. 6:**
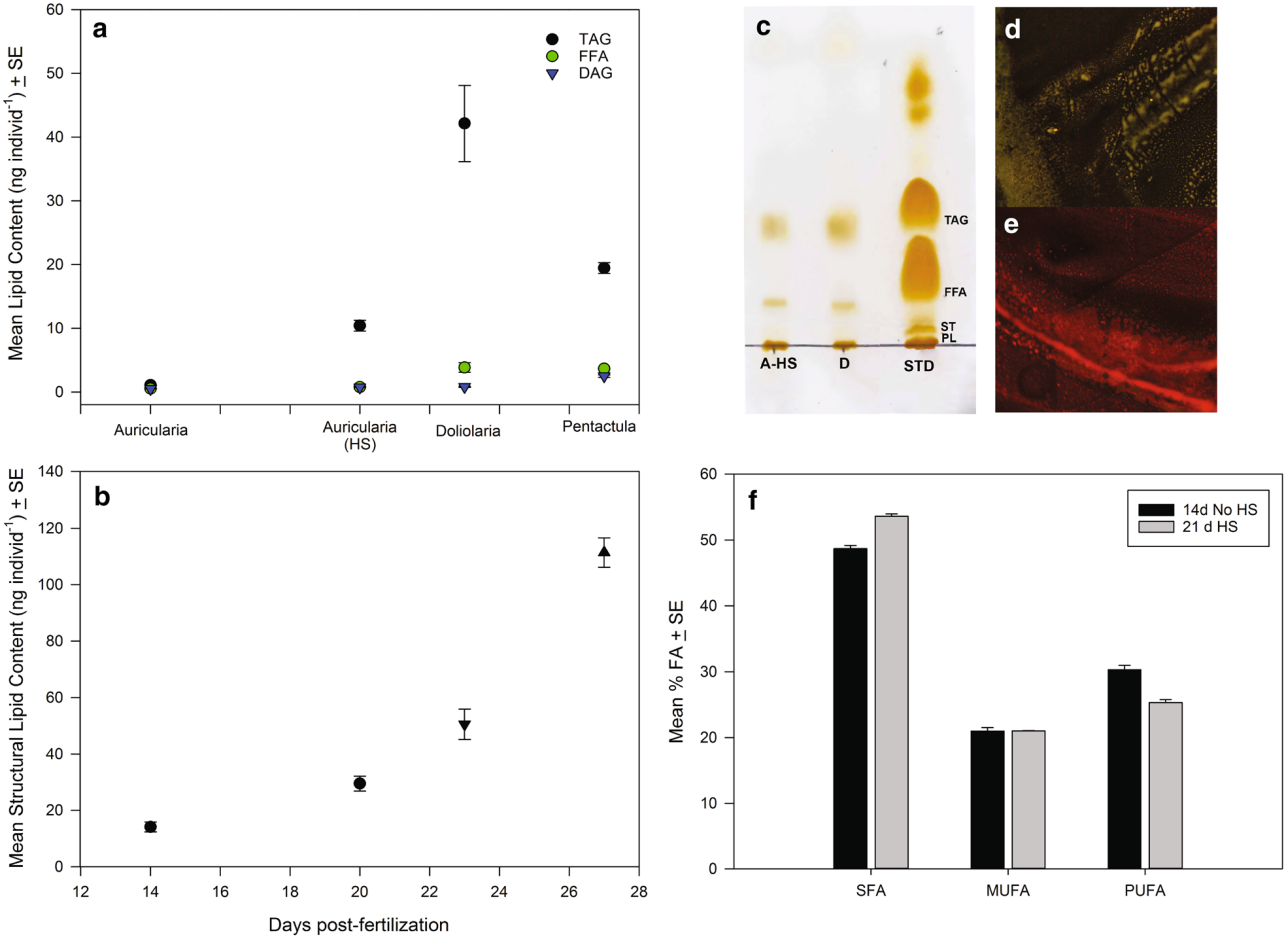
Lipid and fatty acid composition of *A. mollis*. **a** Neutral lipid content of triacylglycerol (TAG), free fatty acid (FFA) and diacylglycerol (DAG) in auricularia, auricularia with hyaline spheres (HS) and through metamorphosis via doliolaria to pentactula. (*N* = 5). **b** Total structural lipids (phospholipids and sterol) as in Panel A, but with age of larvae (days post-fertilization) as X-axis. **c** TLC plate developed in a neutral solvent system containing hexane–diethyl ether–acetic acid (80:20:1, by vol.) followed by staining with iodine vapours. Lipid extracts of *A. mollis* auricularia with HS (A-HS), doliolaria (D) and lipid standards (STD). Grey line shows origin. **d** Re-extracted TAG band from TLC plate stained with Nile Red shows yellow colour under blue light excitation. **e** Re-extracted FFA band from TLC plate stained with Nile Red shows red colour under blue light excitation. **f** Fatty acid composition of auricularia and auricularia with hyaline spheres (HS) divided into saturated (SFA), monounsaturated (MUFA) and polyunsaturated (PUFA) fatty acids (*N* = 3)

Nile Red and LipidTOX™ staining suggests that the HS in auricularia are accumulating large amounts of a neutral lipid from the diet. Using Iatroscan thin-layer chromatography–flame ionization detection (TLC–FID) we found a significant increase in the neutral lipids triacylglycerol (TAG) and free fatty acid (FFA) over the period of HS accumulation in the auricularia (days 14 and 20; Fig. 6a, Table 1). The auricularia-to-doliolaria transition at day 23 results in a non-feeding larva that is almost half the size, the dismantling of the looped ciliated band and extensive remodelling of the hydrocoel and water vascular system (Fig. 4). This transition resulted in a nearly fourfold increase in TAG and FFA, and 1.5 times the amount of structural lipids (phospholipids and sterols) (Fig. 6a, b). As the doliolaria is not able to obtain external sources of neutral lipids, we hypothesize that polar phospholipids made available during the metamorphic transition to the smaller doliolaria larvae are being converted to TAG and FFA, which are then used to fuel the remainder of the metamorphic process. From the doliolaria to pentactula there was intense utilization of the energetic lipid TAG, which halved in only four days (Fig. 6a), a smaller increase in DAG, no significant change in FFA and a further increase in structural lipids associated with differentiation of new structures, such as the external podia and oral tentacles (Table 1).

**Table 1:** Neutral lipid content of triacylglycerol (TAG), free fatty acid (FFA), diacylglycerol (DAG) and structural lipid content (phospholipid and sterol) in auricularia (A), auricularia with hyaline spheres (HS) and through metamorphosis via doliolaria (D) to pentactula (P) (see Fig. 7)

Lipid class	Pseudo-F_3,16_	*p*	Paired tests
TAG	33.20	0.0001	A A-HS D P
FFA	22.33	0.0003	A A-HS [D = P]
DAG	78.45	0.0001	A [A-HS = D] P
Structural	109.89	0.0001	A A-HS D P

One-way permutational analysis of variance (Type III SS, 9999 unrestricted permutations of raw data) using the PERMANOVA + 1 package within PRIMER 7.0.13 (Primer E, Quest Research Ltd) on a Euclidean distance matrix. Pairwise tests show stages that are significantly different separated by spaces; stages that are not significantly different are shown by square brackets

To address the apparent incongruity of the LTC containing a dense aggregation of neutral lipids when stained with the specific neutral LipidTOX™ but indicating a more polar lipid than that contained in the HS when stained with Nile Red (Fig. 1c, d, 2c), we used traditional plate-based TLC to separate the neutral lipid classes (Fig. 6c). Lipid extracts from late auricularia larvae with HS corroborated results from TLC–FID indicating that TAG is the main neutral lipid type present in the larvae along with small amounts of FFA (Fig. 6c). Isolation of the TAG band from the TLC plate, re-extraction and Nile Red staining showed that *A. mollis* TAG fluoresced yellow (Fig. 6d), while the more polar FFA fluoresced red (Fig. 6e). This suggests that the LTC are transporting FFA, which are then converted to TAG within the HS.

Interestingly, comparison of the % FA composition of auricularia without HS (14 days) and with HS (21 days) showed a significant increase in saturated fatty acids (*t*_4_ = −8.24, *p* = 0.0011), a significant decline in polyunsaturated fatty acids (*t*_4_ = 6.35, *p* = 0.0032) and no change in monounsaturated fatty acids (*t*_4_ = −0.01, *p* = 0.992, Fig. 6f). Further, the TAG separated by TLC from the late auricularia stage (as in Fig. 6c) had a higher SFA content (64.9%, *N* = 3) when compared to whole larvae with HS (53.6%, Fig. 6f). This suggests that the conversion of FFA within the LTC to TAG within the HS resulted in a change in saturation of the component fatty acids.

## Discussion

**Fig. 7:**
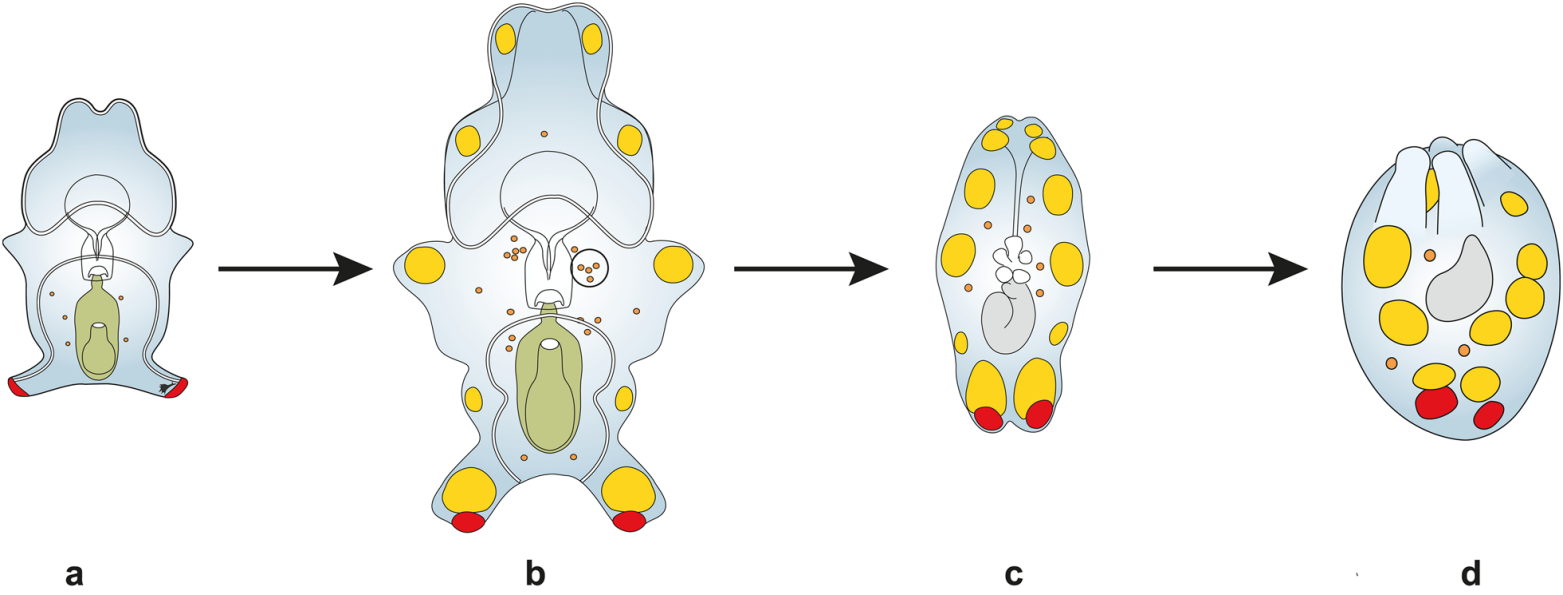
Summary diagram of size and arrangement of lipid transporting cells (LTC, orange circles), hyaline spheres (HS, yellow) and granular masses (GM, red) during larval development and metamorphosis of *A. mollis.* Green colour of digestive system indicates active feeding on phytoplankton and lipid accumulation. Grey colour of digestive system indicates lipid use during the non-feeding period. Images of: **a** early auricularia, **b** late auricularia, **c** doliolaria and **d** pentactula; drawn at the same scale based on photographic images. In the early auricularia the GM and a few small LTC are observed. The late auricularia is a much larger larva, with an increase in the number and size of the LTC (one group circled) that have transported neutral lipids to multiple HS in the base of the larval arms. During the metamorphic transition to the doliolaria the HS release LTC to fuel the reorganization of the gut and the development of the water vascular system (primary tentacles shown in white). In the pentactula the primary tentacles are now external and HS remain in the juvenile stage to provide further nutrients for the peri-metamorphic period. The function of the GM remains unknown

Here, using a multifaceted approach (living material, histological sections, lipid-specific dyes, chromatography) we have resolved the long-standing enigma of the function of the hyaline spheres in sea cucumber larvae, showing that HS have an important nutritional role in late larval development and during metamorphosis in *A. mollis*. Neutral lipids (TAG, FFA) derived from the phytoplankton diet are transported by LTC from the walls of the stomach and intestine through the blastocoel to the HS (Fig. 7). This means that the lipid stores are spatially separated from the major morphogenic events during the metamorphic transition from auricularia–doliolaria–pentactula (settled juvenile, Fig. 7). Nearly 20 ng of neutral lipids remained per pentactula at the end of these experiments (27 days) which would provide a generous “buffer” for metabolic costs until the juvenile has a fully functioning digestive system (i.e. through the peri-metamorphic period).

In addition to confirming a nutritional role of the HS we have shown the mechanism whereby nutrients are transported from the digestive system of auricularia larvae, a significant gap noted in previous studies [7, 15]. We first identified and named the potential transport cells blastocoelic spherules (BS), based on their location, shape and visible movements under light microscopy. Later, through the use of lipid-specific dyes, fluorescent/confocal microscopy and plastic sections, we were able to propose that BS and LTC are the same cell type and that the LTC actively move through the blastocoelic cavity of the auricularia to deliver lipids to the HS.

It is important to note that the BS/LTC, whose function is described here, have been morphologically identified in previous studies on sea cucumber development, for example the “vesiculated mesenchyme cells” in the histological sections of Balser et al. ([27]; their Figs. 5, 6), with one image clearly showing the association with the intestinal wall. Similarly, the light microscope and TEM images of Dautov ([16]; his Fig. 1) show “amoebocytes” that are present in the blastocoel, closely associated with the HS, and the histological sections of Dolmatov et al. [21] showing “mesenchyme cells” in the blastocoel. In addition, both Dautov [16] and Hörstadius [18] show amoebocytes/mesenchymal cells within the HS as we have shown here. Finally, the role of blastocoelic cells in the utilization of HS material in the doliolaria and juvenile stage is depicted in TEMs by Lacalli and West ([28], their Fig. 4e) as “cytoplasmic masses”, although they propose that these are derived from the larval gut and not the HS. These previous studies provide additional support for the proposed mechanism for transport of nutrients within the blastocoel using secondary mesenchyme cells.

The movement of mesodermal cells within the blastocoel of larval echinoderms has been known since Metchnikoff [29] described amoeboid mesodermic phagocytes collecting around wounds or foreign objects in starfish bipinnaria in 1893. More recent work has described a range of secondary mesenchyme cells in the echinoderm blastocoel including skeletogenic, myogenic, phagocytic, immune cells and pigment cells (e.g. [30–32]). Rather than a fluid-filled space, the gel-like blastocoel allows the free movement of mesenchyme cells [33] and is fundamental both to the immune response [30–32] and, as shown here, to the free movement of nutrients from one part of the larva to another.

Holothurians are, however, unusual among planktotrophic echinoderms in transporting lipid reserves obtained from the phytoplankton food, which typically accumulates in the stomach wall in echinoids [3, 6] and asteroids [4]. A notable exception, however, is the ophiopluteus of *Ophiocoma pumila* where an “oily accumulation” forms within the posterior tip of the ophiopluteus beneath the ciliary band [34]. This species metamorphoses through a vitellaria stage, similar in form to the sea cucumber doliolaria in possessing ciliated bands and, as in the sea cucumber larvae seen here, the “oily accumulation” becomes incorporated into the posterior of the vitellaria and is probably used “to sustain the vitellaria” during the exploratory phase before settlement [34]. It would be of considerable interest to use lipid-specific dyes in this, and other ophiuroids with a vitellaria stage, to confirm that the “oily accumulation” contains lipids and whether ophiuroids have a similar cell type to the LTC involved in lipid transport.

A significant advantage of positioning the lipid stores in the periphery of the larva is that it does not interfere with the process of morphogenesis in sea cucumbers and ophiuroids with a vitellaria [7, 9]. In echinoids and ophiuroids, which typically form the rudiment on the left side of the larvae, lipid stores in the stomach wall are spatially separated from the site of rudiment formation [9]. Lipid redistribution to facilitate morphogenesis is also seen in lecithotrophic asteroids, where lipids are shunted to the basal cytoplasm in epithelial cells, and in *Patiriella pseudoexigua*, the species with the greatest lipid load, lipids are also secreted into the embryonic blastocoel where they are stored for the peri-metamorphic period [35, 36]. Strategies for relocation of lipids to facilitate morphogenesis and then to store nutrient reserves for the juvenile may be more widely utilized than currently appreciated in both planktotrophic and lecithotrophic echinoderms, perhaps even in other larvae with gel-filled blastocoels such as the hemichordate tornaria.

Energetic lipid stores in *A. mollis* are primarily TAG, which are accumulated from the phytoplankton diet during the feeding larval stage for metamorphosis, as in other echinoderms (e.g. [3, 4, 37–39]). Our data suggest that the LTC are transporting a lipid that is more polar than TAG, tentatively identified as FFA, although the red staining of LTC with Nile Red could also indicate a hydrophobic protein or lipoprotein [25, 26]. This substance is transported from the epithelial tissues of the stomach and intestine and is transformed to TAG, by processes currently unknown, within the HS. Late auricularia with HS also have a higher percentage of saturated fatty acids, dominated by palmitic acid C16:0, than auricularia that lack HS. SFAs with 10 or more carbons have high melting points (> 29 °C, Ref. [40]). As the TAG within the HS have a high saturated fatty acid content, this might also explain the gel-like nature of the HS, which have been described as soft, malleable and denser than sea water [7, 12, 15–18].

Confirmation that the HS are accumulating and redistributing lipids during the process of metamorphosis in *A. mollis* does not provide the last word on the importance of the hyaline spheres to sea cucumber metamorphosis. Depending on the sea cucumber species, successful metamorphosis can occur in cultured larvae that lack HS [22], there can be a relationship between the number and size of the HS, the phytoplankton diet, and settlement size and survival [41], and some larvae that have large HS do not metamorphose [42]. Whether the HS are a prerequisite for successful juvenile development in all holothurians, or they are only formed under particularly nutrient-rich conditions, as suggested by Smiley et al. [7], is a question that remains unanswered. We also remain perplexed by the role of the granular masses during metamorphosis.

Future research needs to consider whether HS are a prerequisite for metamorphosis in *A. mollis*. We successfully manipulated the size and number of HS through alterations of the phytoplankton diet fed to auricularia (JPD, MAS, unpub. data), but did not follow these cultures through to metamorphosis. Such experiments could prove valuable in assessing the generality of the observation of Smiley [22] in *Apostichopus californicus* that metamorphosis can proceed without HS. Recent transcriptome studies on the auricularia–doliolaria metamorphic transition in the Japanese sea cucumber *Apostichopus japonicus* have also shown KEGG enrichment of over 80 genes involved in the “fat digestion and absorption” category [43]. Detailed examination of these genes, coupled with in situ hybridization showing where in the larval stages these genes are expressed, seems to also offer a fruitful pathway to better understand the nutritional physiology of sea cucumber development.

Finally, the relationship between the LTC and other secondary mesenchyme cells within the blastocoel of the auricularia needs to be examined in more detail. Mesoderm-derived immune cells have been classified into four types in sea urchin larvae [30, 31, 44], and in a single type in asteroid larvae, which is functionally equivalent to that in sea urchins [32, 44]. Recent gene expression [45, 46] and antibody studies [47] have confirmed that sea cucumber larvae have an immune system, although morphological descriptions and detailed microscopy are not yet available for larvae during development and metamorphosis.

## Conclusion

Here we definitively show that the HS in *A. mollis* larvae are storing neutral lipids for use during metamorphosis, i.e. they have a nutritional function. We also describe a secondary mesenchyme cell, the LTC, which moves lipids from the digestive system to the hyaline spheres during late larval development. Although the hyaline sphere itself appears to be unique to sea cucumbers, we hypothesize that the LTC will be found more generally in invertebrate larvae with a gel-filled blastocoel, including other echinoderms and the hemichordate tornaria.

## Methods

*Australostichopus mollis* larvae were obtained from 4 L rearing tanks of 1-µm-filtered sea water (FSW) set-up with gentle aeration under constant light and temperature conditions (12:12-h light:dark cycle at 19 ± 0.5 °C) as outlined in detail in Peters-Didier and Sewell [24]. Larvae were fed a 1:1:1 diet of microalgae *Isochrysis galbana, Dunaliella tertiolecta* and *Chaetoceros muelleri* (CSIRO Australia) supplied at a final concentration of 3000 microalgae cells per ml twice daily. *A. mollis* larval samples were obtained at the auricularia pre-hyaline spheres (14 days), late auricularia with HS (20 days), doliolaria (22 days) and pentactula/juvenile (24–27 days) stages for microscopy and lipid analysis.

Larvae were stained with Hoechst 33342 nuclear stain (Life Technologies) followed by Nile Red (Sigma): 1 µl of 1000× Hoechst 33342 dye was added to 1 ml of larval suspension (~ 40 larvae ml^−1^) in autoclaved FSW, incubated in the dark for 30 min at room temperature and rinsed 3× with autoclaved FSW after gentle centrifugation (2000 rpm, 1 min). One part of Nile Red stock solution consisting of 1:1000 dilution of Nile Red in acetone was then added to 500 parts of autoclaved FSW containing larvae and incubated for 1 h at 4 °C in the dark with occasional gentle inversion. Samples were rinsed in autoclaved FSW, immobilized in circular wells (200 µm deep × 2 mm diameter) on a transparent non-fluorescent Perspex slide and photographed with a Nikon 500 digital sight-cooled colour camera on an epifluorescence microscope (Leica DMR upright) fitted with two long-pass filters: I3 (Ex 450–490 nm, D 510, Em > 515 nm) and A (Ex 340–380 nm, D400, Em > 425 nm).

For confocal microscopy, larvae were stained with LipidTOX™ Red phospholipid stain (Life Technologies) followed by Hoechst 33342 nuclear staining (Life Technologies) and LipidTOX™ green neutral lipid staining (Life Technologies). Two microlitres of 1000× LipidTOX™ Red phospholipidosis detection reagent was added to 1 ml of larval suspension in autoclaved FSW (~ 40 larvae ml^−1^), left in the dark at room temperature for 48 h and rinsed 3x with autoclaved FSW after gentle centrifugation (2000 rpm, 1 min). The larvae were then stained with Hoechst 33342 dye as outlined above, and then 2 µl of 1000X LipidTOX™ green neutral lipid stain was added to 1 ml of the larval suspension and incubated for 30 min. Larvae were visualized in a Andor revolution XD confocal microscope set with a iXon^EM^ (Andor™) camera attached to a Nikon Eclipse-Ti inverted microscope. Three laser channels were used: blue (Ex 405 nm, Em 465 ± 15 nm), green (Ex 488 nm, Em 525 ± 20 nm) and red (Ex 561 nm, Em 617 ± 36.5 nm). Images were processed using Imaris 7 (Bitplane) software. Unstained larvae were used as controls for all observations.

Samples of sea cucumber larvae were fixed and embedded in epoxy resin following the glutaraldehyde and cacodylate buffer method in Byrne et al. [35]. Semi-thin plastic sections (2 µm) were prepared on a Leica EM UC6 ultra-microtome, dried onto factory-coated glass slides (Superfrost^*®*^ Plus, Menzel-Gläser*)* and stained using a polychromatic staining method consisting of methylene blue-azure II and basic fuchsine [48]. Through this method, cytoplasm stains blue, nuclei stain darker blue, collagen, mucus and elastin are pink to red, and fat or intracellular lipid droplets are grey to green [48]. Semi-thin sections were viewed in a Leica DMR upright microscope and photographed, and images analysed with AnalySIS^®^ 5 (Life Science).

Lipid extraction and analysis using Iatroscan thin-layer chromatography–flame ionization detection (TLC–FID) was performed as described in Peters-Didier and Sewell [24] on larval samples (14 days, 20 days, 22 days; *N* = 200 individuals) and pentactula/juveniles (*N* = 60). Traditional TLC was also conducted on lipid extracts (45) from auricularia pre-HS (14 days) and with HS (20 days, *N* = 1000 individuals). Extracts (3 µl) were applied to pre-activated (100 °C for 10 min) aluminium-backed TLC plates (Reveleris, Grace) alongside 1 µl aliquots (10 mg/ml) of the highly purified lipid standards phospholipid (PL: L-α-phosphatidylcholine), free sterol (ST: cholesterol), free fatty acid (FFA: palmitic acid) and triacylglycerol (TAG: tripalmitin). TLC plates were developed in a neutral solvent system containing hexane–diethyl ether–acetic acid (80:20:1, by vol.) and visualized using iodine vapour. Sample bands of TAG and FFA were scraped off the TLC plate, the lipids re-extracted using the Bligh and Dyer [49] method, and silica gel beads removed by centrifugation (3500 RPM for 10 min). The supernatants were dried in instrument-grade nitrogen, re-dissolved in 10 µl of chloroform and the TAG and FFA extracts spotted adjacent to each other on onto Superfrost^*®*^ Plus factory-coated glass slides (Menzel-Gläser). Nile Red staining was in FSW for 30 min at the same concentration used to stain live larvae. Re-extracted lipids were observed under fluorescence microscopy using an I3 filter (Ex 450–490 nm, D 510, Em > 515 nm) and photographed using a Nikon 500 digital sight-cooled colour camera and images analysed with AnalySIS^®^ 5 (Life Science).

Fatty acid methyl esters (FAME) were prepared using the method of Lepage and Roy [50] from total lipid extracts of 5000 *A. mollis* larvae with and without hyaline spheres (*N* = 3) using C19:0 and C23:0 as the internal standards. FAME were separated and quantified using a gas chromatograph (GC 7890 Agilent system) equipped with a mass spectrometry detector (MSD 5975c) as described in Zárate et al. [51]. FAME peaks were identified by comparing their retention times with those of the 37 authentic FAME standards (Supelco Inc.); those not present in the standard mix were compared with those from the National Institute of Standards and Technology mass spectra library (NIST MS Search 2.0), together with the Lipid Library [52]. FA abundances were normalized by C19:0 and grouped into saturated FAs (SFAs: those FAs with simple covalent C–C bonds in the hydrocarbon chain), monounsaturated FAs (MUFAs: those FAs with only 1 double covalent C–C bond in the hydrocarbon chain) and polyunsaturated FAs (PUFAs: those FAs with 2 or 3 double covalent C–C bonds in the hydrocarbon chain).

Statistical analyses were conducted using one-way ANOVA with SAS 9.4 or when assumptions of ANOVA were violated by permutational analysis of variance (Type III SS, 9999 unrestricted permutations of raw data) using the PERMANOVA + 1 package within PRIMER 7.0.13 (Primer E, Quest Research Ltd) on a Euclidean distance matrix.
